# Paracoccidioidomycosis: An uncommon cause of discitis in lupus

**DOI:** 10.1590/0037-8682-0191-2024

**Published:** 2024-07-29

**Authors:** Simone Appenzeller, Lilian Lavras Costallat, Marcelo de Carvalho Ramos, Samuel de Oliveira Andrade, Fabiano Reis

**Affiliations:** 1 Universidade Estadual de Campinas, Departamento de Ortopedia, Reumatologia e Traumatologia, Campinas, SP, Brasil.; 2 Universidade Estadual de Campinas, Departamento de Clínica Médica, Campinas, SP, Brasil.; 3 Universidade Estadual de Campinas, Departamento de Anestesiologia, Oncologia e Radiologia, Campinas, SP, Brasil.

**FIGURE 1:**
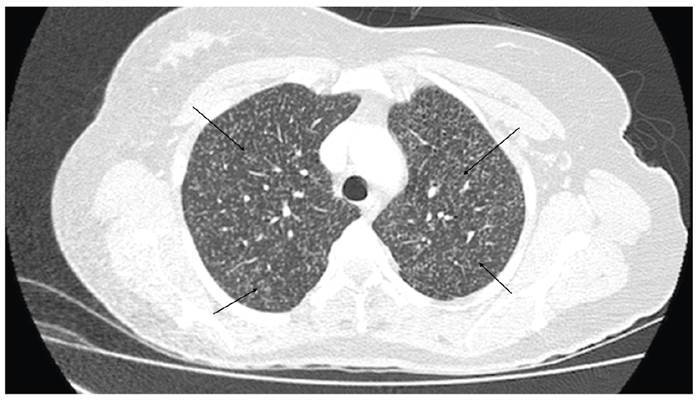
Chest computed tomography, with the axial pulmonary window showing multiple randomly distributed pulmonary micronodules (arrows) in both lungs.

**FIGURE 2:**
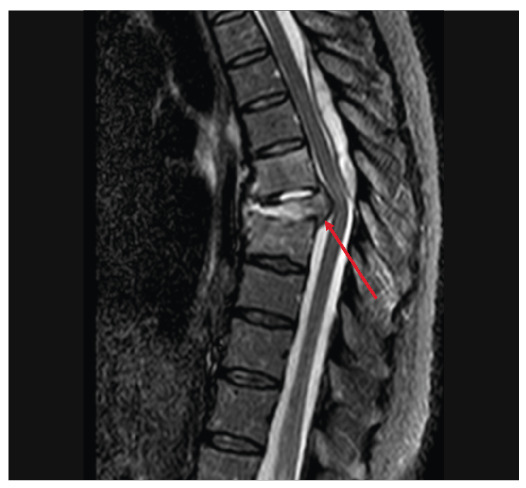
Magnetic resonance imaging. Sagittal T2 short tau inversion recovery showing the hyperintensity of the T6-T7 intervertebral disk and the absence of the internuclear cleft. Hyperintensity of the adjacent vertebral bodies was also observed. The lesion posteriorly compressed the dural sac and the spinal cord (arrow).

**FIGURE 3:**
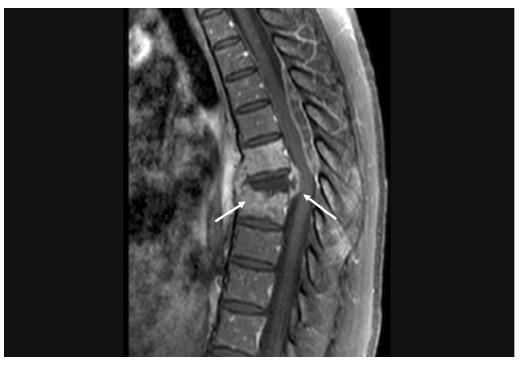
Magnetic resonance imaging. Sagittal contrast-enhanced T1-weighted fat-suppressed MR weighted image of the thoracic spine showing paravertebral collections with peripheral enhancement, enhancement of the T6 and T8 vertebral bodies (arrow), a necrotic collapsed T7 vertebral body, and a posterior epidural mass with peripheral enhancement at the T4-T7 levels, suggesting epidural abscess. An anterior epidural collection at the T7 level combined with angular kyphosis resulted in spinal cord compression (arrow).

A 41-year-old woman with systemic lupus erythematosus (SLE) presented to the outpatient department with progressive 3-month interscapular pain that was refractory to analgesics. The patient’s medications included azathioprine 50 mg, hydroxychloroquine 400 mg, and prednisone 5 mg daily. She reported a weight loss of 7 kg in the previous 2 months but denied experiencing fever or any history of trauma. She lived in a rural area and worked in the agricultural sector. Thoracic spinal radiographs obtained during the initial assessment revealed T7 vertebral collapse and diffuse nodular pulmonary opacities. The patient was hospitalized; chest computed tomography (CT) revealed the presence of diffuse micronodular disease **(** Figure 1) and vertebral collapse associated with signs of spondylodiscitis at T6-T7 and T7-T8, which were confirmed by magnetic resonance imaging (Figures 2 and 3)**.** A T8 paravertebral computed tomography-guided biopsy confirmed the presence of *Paracoccidioides* spp. The patient was treated with itraconazole 100 mg daily for 3 months, resulting in the complete resolution of infection and a good outcome. 

Active disease and immunosuppressive therapy are risk factors for infection in patients with SLE^1^. Invasive fungal infections usually occur in immunocompromised hosts, with the most common pathogens in SLE patients being *Candida albicans, Cryptococcus neoformans,* and *Aspergillus* spp.^2^ ^,^ ^3^. Osteoarticular involvement in paracoccidioidomycosis is unusual, mainly affecting the bones of the chest wall (ribs, sternum, scapula, and acromion). Discitis-osteomyelitis, particularly when caused by fungi, is a rare infection^4^. To our knowledge, only two patients with vertebral body paracoccidioidomycosis have been reported in the literature^5^. 
